# Black Swan Pandemic and the Risk of Pilot Suicide

**DOI:** 10.3389/fpubh.2020.573006

**Published:** 2020-10-29

**Authors:** Alpo Vuorio, Robert Bor

**Affiliations:** ^1^Mehiläinen Airport Health Care Centre, Vantaa and University of Helsinki, Helsinki, Finland; ^2^Centre for Aviation Psychology, Royal Free Hospital, London, United Kingdom

**Keywords:** aviation, pilot, mental health, suicide, COVID-19, unemployment

## Summary

The medical risks to pilots, whether to their physical or mental health, are clearly theoretically raised during the COVID-19 pandemic for the reasons outlined in this paper, and access to medical and psychological support should be improved in order to address pilot stress, distress and the potential for increased pilot suicides as a direct result of economic effects of the pandemic.

The links between pilot suicides and social change, such unemployed threats and financial recession, have not been studied. Significant and sudden changes in society may increase suicide risk and serious mental health problems may affect pilots equally. After the 9/11 terrorist attacks, for example, the risk of suicide by aircraft in the year following the attack was almost four times the average risk in the 5 years prior to the terrorist attack. This paper discusses the potential causes of mental health problems to pilots resulting from COVID-19.

Pilot aircraft-assisted suicide in commercial aviation is a rare phenomenon. In general aviation in the United States, pilot aircraft-assisted suicide rates in a 20-year period was positively determined in 0.33% (24/7,244) of fatal aircraft accident cases (1). Pilot murder-suicides, where it has been concluded through post-accident analysis and investigation that the pilot deliberately crashed a commercial aircraft killing the pilot and all others on board have occurred in six instances over the past 30 years (2). The most recent of these was the Germanwings pilot murder-suicide crash in 2015 on a commercial flight between Barcelona and Dusseldorf (3), an incident which brought this phenomenon to public attention and led to the regulator, the European Aviation Safety Authority, to require that airline pilots in the EU be psychologically assessed prior to joining an airline and for crew to have access to pilot peer support programmes. The regulator also required aviation medical examiners to focus greater attention on pilot mental health and well-being in their annual pilot assessments (4). It is noteworthy that the pilot of the Germanwings aircraft suffered with financial problems in addition to other significant mental health problems.

The links between pilot suicides and social change, such unemployed threats and financial recession, have not been studied, given the low numbers of pilot suicide cases as well as the unpredictability and infrequency of recession, coupled with methodological challenges such as suitable comparison groups and the absence of baseline measures. However, it has been found that significant sudden changes in society may increase the number of pilot suicides. For example, after the 9/11 terrorist attacks in New York, the risk of suicide by aircraft in the year following the attack was almost four times the average risk in the 5 years prior to the terrorist attack (5). Although we are unable to precisely determine a causal link between societal changes on pilot suicide, it is arguably feasible that significant, sudden and adverse changes in society can have an impact on pilot mental health. Due to the deleterious effects of COVID-19 on society generally, and on aviation specifically, we are living through the most significant and enduring aviation crisis in the history of modern commercial aviation. Thousands of air crew worldwide including pilots and cabin crew, are threatened with redundancy, unwelcome changes to their employment contracts such as increased duty times and lower pay, and the prospect that some may never fly for a living again. Currently a large proportion of pilots are furloughed or out of work. As they find employment, it is very likely that their job demand and workload will increase. Additionally, some pilots are self-employed and continue to work on zero-hours contracts and are without employment protection or health care support.

A study of 424 pilots over 35 years ago has shown that during an occupational dispute, the stress experienced by pilots had a significant impact on their mental health (6). This study also demonstrated that the combination of factors including career development, autonomy at work, organizational climate and family support and cohesion are important regarding job satisfaction, but also potentially increase the risk of accidents if work and personal stress levels are left unchecked. It has been shown that economic crises increase the mental burden on work and workload (7). In addition to this, it is well established that an economic downturn is associated with an increase in suicides in the general population (8, 9).

The social, economic, employment and personal challenges of COVID-19 in society threatens several factors important for pilots' mental well-being. The current situation is compounded by the additional social stress brought about by social and physical distancing measures brought in to contain the spread of infection. A recent position paper by the UK Academy of Medical Sciences offers a strategy for how to study and to take account of the psychological, social and neuroscientific aspects of the pandemic (10). It is recognized that the pandemic may increase suicide rates just as Acute Respiratory Syndrome (SARS) did in 2003 (11–13). This risk is increased due to economic prolonged downturn, which particularly affects aviation. The most significant and concerning effects may only become apparent in the future. Another threat concerns pilots who themselves have suffered with infection with COVID-19. Although infection may be asymptomatic in many instances, it may lead to serious mental and neurological problems in those who have been hospitalized due to the effects of the virus and also side-effects of hospitalization and treatment (10, 14). It has been reported that those people who suffered serious infection with SARS were at increased risk of post-traumatic stress disorder and depression (11, 12). Post-traumatic stress disorder has been shown to be associated with fatal aircraft accidents (15). COVID-19 data from a national sample of over 10,000 U.S. adults gathered on March 2020 showed that about 15% of respondents had high risk on the Suicide Behaviors Questionnaire-Revised survey (16).

One challenge in health care generally, and in aviation medicine specifically, is how to best prevent or mitigate the risk of mental health problems and especially suicide risk during the COVID-19 pandemic whilst airline employees suffer a threat to their job and livelihoods due to economic effects. It is important to note that each time a pilot visits their aeromedical examiner (AME) or aviation psychologist, there may be a fear the loss of their medical certification (17). It has been shown that pilots may be reluctant to report to their AME conditions that could affect crew licensing due to their concerns that this could affect their livelihoods (18). Private medical insurance and support and company-organized occupational healthcare is not available to many pilots. It is not yet possible to determine how physical health, routine medical checks and AME visits by pilots have been affected by the COVID-19 pandemic and it is possible some pilots will have avoided seeking health care during this time for economic, infection risk and occupational threat reasons. Some regulators, such as European Aviation Safety Agency (EASA) have allowed pilots to renew their medical certificates by accessing AME's remotely and by extending the validity of their licenses, reducing contact between airline crew and medical specialists.

The medical risks to pilots, whether to their physical or mental health, are clearly theoretically raised during the COVID-19 pandemic for the reasons outlined in this paper, and access to medical and psychological support should be improved in order to address pilot stress, distress and the potential for increased pilot suicides as a direct result of economic effects of the pandemic. As there are no standardized clinical scales for assessing suicide risk, the focus of support should target all pilots who are distressed, have experienced severe life-events in their own life, have depressive symptoms or express hopelessness (19). Thus, all pilots with any risk should receive psychoeducation, information on stress management and needs-based care. This requires also informing and educating health care staff working with the pilots.

## Author Contributions

All authors listed have made a substantial, direct and intellectual contribution to the work, and approved it for publication.

## Conflict of Interest

The authors declare that the research was conducted in the absence of any commercial or financial relationships that could be construed as a potential conflict of interest.
